# Tracing the impact of maternal communication deviance on joint engagement: The mediating role of maternal supportive behaviors

**DOI:** 10.1002/imhj.70126

**Published:** 2026-11-10

**Authors:** Paulo de Sousa, Rebecca Hartley, William Sellwood, Niamh Grace, Helen Sharp, Jonathan Hill, Richard P. Bentall

**Affiliations:** ^1^ Department of Primary Care and Mental Health, Institute of Population Health, The Eleanor Rathbone Building University of Liverpool Liverpool UK; ^2^ Hartley Hospital Merseycare NHS Foundation Trust Southport UK; ^3^ Division of Health Research, Faculty of Health and Medicine, Furness Building Lancaster University Lancaster UK; ^4^ Department of Child and Adolescent Mental Health, Insights Psychology DMCC The Dome Tower Jumeirah Lake Towers Dubai UAE; ^5^ Faculty of Health and Education, Ormond Building Manchester Metropolitan University Manchester UK; ^6^ Clinical and Applied Psychology Unit, School of Psychology University of Sheffield Sheffield UK

**Keywords:** caregiver sensitivity, communication deviance, early development, joint engagement, maternal scaffolding

## Abstract

Communication Deviance is a disorganized communicative style that can disrupt shared meaning‐making in parent–child interactions and is considered a risk factor for later mental health difficulties. This longitudinal study, conducted in the UK, examined whether maternal Communication Deviance assessed during pregnancy predicted joint engagement and related processes. Participants were 229 mothers (95.9% White British; *M* = 27.6 years, *SD* = 6.1). Maternal Communication Deviance was assessed antenatally, and mother–child dyads were observed during play at 30‐months. Communication Deviance predicted lower total joint engagement, including supported (*β* = –.18, *p* = .007), coordinated (*β* = –.17, *p* = .016), and symbol‐infused joint engagement (*β* = –.24, *p* < .001), and was associated with reduced supportive behaviors (*β* = –.40, *p* < .001) and dyadic attunement (*β* = –.31, *p* < .001). Mediation analyses indicated that supportive behaviors accounted for the associations between Communication Deviance and joint engagement outcomes. These findings suggest that Communication Deviance may constrain children's opportunities for shared attention and meaning making. The study supports developmental models that highlight early communicative processes as foundational to socio‐cognitive and relational development and identifies supportive behaviors as a potential target for interventions promoting early mental health and parent–child interaction.

**Key Findings:**

1. Higher maternal Communication Deviance during pregnancy predicted less Total, Supported, Coordinated, and Symbol‐infused joint engagement at 30 months.
2. Reduced maternal supportive behaviors fully explained the link between Communication Deviance and lower joint engagement and dyadic attunement.
3. Maternal Communication Deviance may act as an early environmental risk factor, highlighting the importance of supporting maternal responsiveness to promote healthy development.

**Statement of Relevance:**

This study examines how maternal Communication Deviance measured during pregnancy predicts joint engagement and dyadic attunement at 30 months, processes central to early socio‐cognitive and relational development. By identifying maternal supportive behaviors as a key mechanism linking Communication Deviance to interactional quality, the research highlights a modifiable target for early intervention. Findings advance understanding of early environmental risk factors and inform strategies to support parent–child interactions, directly addressing foundational processes relevant to child and early childhood mental health.

## 1 COMMUNICATION DEVIANCE AS A RISK FACTOR FOR PSYCHOPATHOLOGY

Communication Deviance refers to a communicative style characterized by vagueness, ambiguity, and disruptions in shared meaning between interlocutors (Singer, 1965a, b; Singer et al., 1978; Tienari & Wahlberg, 2008; Wynne, 1963a; Wynne et al., 1963, 1977b, 1984). It encompasses a range of linguistic and interactional disturbances that can hinder mutual understanding during social exchange (e.g., unclear referents, contradictory statements, or poorly timed contributions).

Communication Deviance has been most extensively studied in the context of schizophrenia‐spectrum disorders, where elevated levels in parents have been associated with increased risk of thought disorder and broader psychopathology in offspring (de Sousa et al., 2014; Roisko et al., 2014). Longitudinal findings indicate that Communication Deviance precedes the onset of such difficulties, supporting its role as an environmental risk factor rather than a response to child characteristics (Goldstein, 1987). However, Communication Deviance is not specific to schizophrenia; similar communicative patterns have been observed in families experiencing other forms of psychopathology, relational distress, and needs, suggesting it is better conceptualized as a dimensional and transdiagnostic construct (Miklowitz & Stackman, 1992).

Evidence from adoption studies further suggests that Communication Deviance interacts with genetic vulnerability, such that its impact on offspring outcomes depends on broader risk context (Wahlberg et al., 1997, 2000). Overall, this body of work supports the view that parental CD constitutes an environmental risk factor in the development of psychopathology. However, the mechanisms through which CD operates in early caregiving contexts remain poorly understood.

## 2 PLAUSIBLE DEVELOPMENTAL MECHANISM

One proposed pathway is through the disruption of early interactional processes in childhood. In particular, Communication Deviance may interfere with joint engagement which refers to episodes of mutual attention, communication, and emotional connection between caregiver and child around a common interest or task (Adamson et al., 2004, 2009, 2012). These interactions are critical for the development of social understanding, emotional regulation, and cognitive skills (Adamson et al., 2019) and the quality of nonverbal and verbal interactions (e.g., dyadic connectedness and fluency) seemed to be important for the child's cognitive and social development (Hirsh‐Pasek et al., 2015).

If caregiver communication is vague, inconsistent, or poorly timed, opportunities for sustained shared attention and meaning making may be reduced. In this way, Communication Deviance may constrain the quality of early interactional experiences that support development. Although this account is theoretically plausible, it has not yet been directly tested.

### 2.1 Joint engagement

Joint engagement refers to sustained, reciprocal interactions between a child and a social partner in which both participants are actively involved in a shared activity and maintain a common focus over time (Adamson, 2018; Adamson et al., 2014; Trevarthen & Aitken, 2001). These engagement states are characterized not only by shared attention, but also by the quality of the interaction, including affective attunement, reciprocity, and the use of gestures, actions, and language. This construct is grounded in sociocultural developmental theory (Vygotsky & Cole, 1978) and early work on infants’ emerging capacity to share attention with caregivers (Scaife & Bruner, 1975).

Within these broader engagement states, joint attention is best conceptualized as a set of social‐communicative skills that enable the coordination of attention between partners, including behaviors such as responding to joint attention, initiating joint attention, gaze following, and pointing (Adamson et al., 2019; Mundy, 2018). Rather than being synonymous with joint engagement, these skills support the initiation, maintenance, and elaboration of joint engagement episodes. Thus, joint attention reflects the child's moment‐to‐moment attentional coordination abilities, whereas joint engagement captures the broader, dynamic context in which these skills are embedded and expressed (Adamson et al., 2019).

Disruptions to both joint attention skills and joint engagement states are well documented in autism and are thought to contribute to broader differences in social communication and cognition (Adamson et al., 2009; Nyström et al., 2019). These early social interactions are supported by developing perceptual‐motor capacities (Butterworth & Jarrett, 1991) and neurocognitive mechanisms that orient the child toward social information (Baron‐Cohen, 1997), but they are fundamentally shaped within relational and affective contexts (Hobson, 2005; Hobson & Hobson, 2011; Racine & Carpendale, 2007).

During the first year of life, infants begin to engage in behaviors that recruit others’ attention, such as gaze following and pointing, with more consistent use emerging toward the end of the first year (Salter & Carpenter, 2025). Across the second year, episodes of joint engagement become increasingly sustained, differentiated, and socially coordinated (Bakeman & Adamson, 1984; Charman et al., 2000; Mundy & Gomes, 1998). These interactions provide a critical context for development, supporting the emergence of social understanding (Mundy, 2018; Tomasello, 2014), language acquisition (Loy et al., 2018; Mundy et al., 2007; Tomasello & Farrar, 1986), cognitive development (Mundy & Newell, 2007; Mundy & Sigman, 2015), and emotion regulation (Morales et al., 2005).

Joint engagement episodes are inherently multi‐modal, integrating gaze, gesture, touch, and language (Schatz et al., 2022; Suarez‐Rivera et al. et al., 2022). Caregivers play a central role in structuring and sustaining these interactions through sensitivity, contingent responding, and scaffolding (Bakeman & Adamson, 1984; Hustedt & Raver, 2002). For example, following the child's focus of attention supports the emergence of coordinated joint engagement, whereas more directive behaviors may characterize supported joint engagement (Mendive et al., 2013). In naturalistic contexts, caregiver involvement, particularly when it is multi‐modal, substantially increases the duration and complexity of play interactions (Schatz et al., 2022). Indeed, caregiver behaviors such as sensitivity, cognitive stimulation, and positive affect are positively associated with both the frequency and duration of joint engagement episodes (Koşkulu et al., 2021), whereas difficulties in affective attunement may reduce the likelihood of initiating shared engagement (Annie Yoon et al., 2014).

Importantly, the quality of parent‐child interaction appears to be a strong predictor of developmental outcomes. For example, dyadic fluency and connectedness during interaction predicts language outcomes beyond the quantity of linguistic input, particularly in socioeconomically disadvantaged contexts (Hirsh‐Pasek et al., 2015). Similarly, early caregiver attunement predicts later joint attention abilities, which in turn mediate associations with executive function development (Brandes‐Aitken et al., 2020). Time spent in joint engagement and caregivers’ responsiveness to the child's attentional focus are also associated with the development of gesture and early language (Carpenter et al., 1998), while infants’ responsiveness to joint attention bids predicts later vocabulary outcomes (Morales et al., 2000).

Across development, joint engagement becomes increasingly symbol‐infused, providing a foundation for language use and meaning‐making (Adamson et al., 2004). By early childhood, these interactions expand to include references to absent objects and events, supporting more complex communicative exchanges about the past and future (Adamson & Bakeman, 2006; Adamson et al., 2014).

## 3 PRESENT STUDY

The study examined whether antenatal maternal Communication Deviance (at 32 weeks’ gestation) predicts reduced Joint Engagement and related interactional processes at 30 months in primiparous mothers. It was hypothesized that higher maternal Communication Deviance would be associated with less time in Joint Engagement states, less Maternal Scaffolding, Symbol Highlighting, Following the Child's focus, less Fluency and Connectedness of communication and Sustainability of Shared topic in the dyad and poorer Expressive Language, and that the relationships between maternal Communication Deviance, Joint Engagement, dyadic attunement variables, and the child's Expressive Language would be mediated by maternal behaviors such as Scaffolding, Symbol Highlighting, and Following the Child's Focus.

## 4 METHOD

### 4.1 Recruitment and sample

The study draws on data from the Wirral Child Health and Development Study (WCHADS; Sharp et al., 2012) which is a longitudinal study that aimed to identify early social, emotional, and biological factors involved in the development of conduct disorder. The study employed a stratified two‐stage cluster design, recruiting a larger sample of primiparous mothers during pregnancy, from which a random subsample was drawn for intensive assessment (*n* = 316). Participants were English‐speaking, aged ≥18, and sought antenatal care at 12 weeks’ gestation between February 2007 and October 2008 in the Northwest of the UK. A detailed flowchart of sampling, recruitment and attrition rates is available elsewhere (Sharp et al., 2012). Only data from the intensive subsample were used, as this included detailed observational data on mother‐child interactions. Of the 316 participants, 29 declined or were unable to complete the 5‐min speech sample task. Of the 287 who provided a 5‐min speech sample task during pregnancy, 230 attended the 30‐months’ assessment involving mother‐child play observations. Reasons for non‐attendance included withdrawal from the study, illness, and other commitments. Participants were required to be English‐speaking, and the sample was predominantly monolingual English‐speaking; however, formal mother‐tongue language status was not systematically recorded.

## 5 PROCEDURE

### 5.1 weeks speech samples (antenatal)

At 32 weeks’ gestation, mothers were invited to provide speech samples about their unborn child (Leeb et al., 1991). The mother was instructed using the following prompt:

“I would like to hear your thoughts and feelings about your baby at the moment, in your own words without me interrupting. When I ask you to begin, I would like you to speak for 5 minutes, tell me what your impressions have been of your baby whilst you've been pregnant. Have you got any questions before we start?”

All interaction samples were reviewed to ensure they reflected continuous, naturalistic engagement; no minimum word‐count threshold was set a priori for inclusion.

### 5.2 Communication Deviance

Communication Deviance was scored with the Interactional Communication Deviance scoring scheme which was originally developed for family interaction (Velligan, 1985). The scheme focuses on eight types of communication disturbances derived from research on Communication Deviance (Doane & Singer, 1977; Singer, 1965a, b; Wynne, 1963a,b; Wynne et al., 1977, 1963b): (1) abandoned, abruptly ceased, uncorrected remarks, (2) unintelligible remarks, (3) contradictions, denials and retractions, (4) ambiguous referents, (5) extraneous questions and remarks, (6) tangential, inappropriate responses to questions or remarks, (7) odd word usage/odd sentence construction, and (8) reiteration. Code examples are available in a previous publication (de Sousa et al., 2019) and in Table 1.

**TABLE 1:** Definitions and examples of the Communication Deviance codes (*Velligan*, 1985 *)*.

Communication Deviance code	Definition	Example
Abandoned, abruptly ceased, uncorrected remarks	Speaker abruptly abandons an idea without returning to it leaving a sense of no closure.	*“M: You know, what does it…I wanna look like that you know. So it wasn't…That's, I think that's what was sort of so err, hard.”*
Unintelligible remarks	Speaker makes remarks that are not understandable in the context of conversation.	*“M: At the moment I feel like…‘cause even, we had a doctors appointment yesterday morning and we still can't categorically say we know a lot about genetically what happens, what the baby's made of so I don't think many people know that you see.”*
Contradictions, denials and retractions	Speaker contradicts, openly retracts or denies what he has previously said.	*“M: That's all really, I'm just happy about it (…) M: I don't know how I feel.”*
Ambiguous referents	Speaker uses linguistic referents that are unclear or ambiguous and that could be referring to more than one person or object.	*“M: I maybe don't allow myself as much of that as what maybe I should do because I'm always focussed on making sure everything's okay, you know.”*
Extraneous questions and remarks	Speaker makes comments or asks questions that are extraneous to the task.	**“** *M: What do people normally say? M: It's very strange being asked to ramble”*
Tangential, inappropriate responses to questions or remarks	Speaker makes non‐sequitur replies to questions or remarks.	*“(…) Err, chest of drawers and we just need to get a little wardrobe and I've got like this lamp, a Winnie the pooh lamp, that plays music and stuff and you can get like a Winnie the Pooh thing to put over the cot and stuff, make it all dead nice. It doesn't have to be Winnie the Pooh but I thought Winnie the Pooh would be nice, plus [partner's name]’s mum gave us some Winnie the Pooh pictures for the walls so that's made us decide Winnie the Pooh.“*
Odd word usage/odd sentence construction	Speaker uses of words or sentences in a way that is odd, incorrect or out of context.	“*M: I feel like quite protective over her even though she's not here already.”*
Reiteration	Speaker repeats the same thought, idea or word several times without adding new information.	*“M: I think I probably worry probably as a tendency more than probably most people would but then that's probably because I probably am aware of every eventuality.”*

The scheme demonstrates good reliability and construct validity (Velligan et al., 1990) and has been applied in clinical (Velligan et al., 1995, 1996), high‐risk (Velligan et al., 1988) and natural speech samples (Docherty, 1993), including 5‐min speech samples (Kymalainen & Weisman de Mamani, 2008; Kymalainen et al., 2006).

The Communication Deviance raw score reflects the total frequency of coded Communication Deviance instances across the eight categories. The Communication Deviance ratio was calculated as: (number of Communication Deviance instances ÷ total word count) × 100, providing a rate‐adjusted index of Communication Deviance. The minimum possible score is 0, with no fixed maximum value, as this depends on speech length and frequency of Communication Deviance occurrences (Hirsch & Leff, 1971, 1975).

The speech samples were audio‐recorded and transcribed by the WCHADS team, then coded for Communication Deviance. The period of training involved reading relevant papers in the Communication Deviance field (Singer & Wynne, 1966), the scoring manual (Velligan, 1985), and regular meetings between raters and communication exchanges with other researchers in the field. Raters were blind to any background information about the participants using anonymized transcripts and audio‐recordings. Coders were trained to distinguish dialectal variation from Communication Deviance by focusing on functional communicative clarity rather than conformity to Standard English. Non‐standard grammatical forms or regional linguistic features were not coded as Communication Deviance unless they resulted in ambiguity, disruption of referential coherence, or breakdown in shared meaning within the speech sample. The first author completed all primary coding, and a second independent coder completed reliability coding on approximately 10% of transcripts. The second coder was a local resident of the region, providing contextual familiarity with regional speech patterns. Any discrepancies between coders were resolved through discussion and reference to the coding manual. Some of the categories were very infrequent (e.g., reiteration), but the inter‐rater reliability was good (the intraclass correlations for the items ranged from .77 to .97).

### 5.3 30 months play protocol

The mother and child were first presented with three cloth bags numbered 1, 2, and 3, which were left on the floor of the room in order and close to the mother. This structured play procedure is consistent with standardized observational parent‐child play paradigms, such as the “three‐box” task used in the NICHD Early Child Care Study, which provide a standardized context for observing parent‐child interaction across dyads. Although the three‐box task was not developed specifically to assess joint engagement, it similarly offers comparable opportunities for shared attention and coordinated interaction. The mother was then given the following prompt from the research assistant:

“This activity will take about 10 minutes. We would like for your child to spend some time with each of the three toys in the bags. Please have [child] start with the #1 bag and finish with the #3 bag. During this activity, you may play with [child] if you would like. Do you have any questions before we get started?”

Each cloth bag contained a toy (a storybook, a toddler kitchen and a toy house, respectively). At the start, the mother was informed that the sound of a baby crying would be played for 30 seconds during the 10‐min session. This element formed part of the broader WCHADS protocol designed to assess maternal responses to child's distress and was included for all participants under standardized conditions. The present study did not analyze responses to this stimulus. Given its brief duration and uniform administration across participants, this component is unlikely to have systematically influenced the associations examined in the present analyses. After 10 min, the mother was told the task had ended by the researcher calling for “tidy‐up time”. The play was captured by four cameras, providing four angles of the interaction.

### 5.4 Joint engagement

Joint engagement was coded using the Joint Engagement Rating Inventory (JERI; Adamson et al., 2004; 2009; 2012), a 7‐point observational rating system designed to assess the quality and quantity of caregiver–child engagement during interaction (Adamson et al., 2012). The JERI includes multiple dimensions of child engagement, caregiver behavior, child behavior, and dyadic interaction during structured play. A brief description of each selected item is provided in the text, and the full definitions and scoring anchors are presented in Table 2.

**TABLE 2:** Full description of interaction ratings (Adamson et al., 2004; 2009; 2012).

Rating item	Definition	Rating anchors		
		1 =	4 =	7 =
Total Joint Engagement	Total Joint Engagement refers to the overall amount of time a child is actively sharing attention with a caregiver around the same object, event, or topic during an interaction.	No episodes of joint engagement.	The child is in joint engagement for approximately ½ of the recording, displaying several brief or a few relatively sustained episodes.	The child is in joint engagement for almost all of the recording.
Supported Joint Engagement	Supported Joint Engagement refers to periods of triadic engagement in which the child and caregiver are jointly engaged with a shared object or topic, and the caregiver plays an influential role in supporting and shaping the child's experience. The child does not actively coordinate attention between the shared object and the caregiver (e.g., through frequent gaze shifts, gestures, or communicative bids), although both partners remain engaged in the same activity.	No episodes of Supported Joint Engagement.	Caregiver and child spend about ⅓ of the recording in Supported Joint Engagement that is of moderate quality or briefer episodes that are of striking quality.	Caregiver and child are frequently in rich and varied episodes of Supported Joint Engagement.
Coordinated Joint Engagement	Coordinated Joint Engagement refers to periods when a child and caregiver are focused on the same object or activity, and the child actively coordinates attention between the object and the caregiver (e.g., by looking back and forth, gesturing, or communicating).	No episodes of Coordinated Joint Engagement.	Caregiver and child spend about ⅓ of the recording in Coordinated Joint Engagement that is of moderate quality or briefer episodes that are of striking quality.	Caregiver and child are frequently in rich and varied episodes of Coordinated Joint Engagement.
Symbol‐infused Joint Engagement	Symbol‐infused Joint Engagement refers to periods when a child and caregiver share attention to an object, event, or topic, and the child uses symbols—such as words, signs, or pretend play—to represent and communicate about that shared focus.	No episodes of Symbol‐infused Joint Engagement.	Caregiver and child spend about ⅓ of the recording in Symbol‐infused Joint Engagement that is of moderate quality or briefer episodes that are of striking quality.	Caregiver and child are frequently in rich and varied episodes of Symbol‐infused Joint Engagement.
Scaffolding	Scaffolding refers to the caregiver's use of supportive strategies that help the child participate in, sustain, and extend interaction, play, or communication. The caregiver adjusts the level of help depending on what the child can currently do, providing just enough assistance to keep the activity going while also gently encouraging more advanced or complex behavior.	Caregiver provides minimal support for the child's communication and/or actions and objects.	Caregiver provides moderate levels of support.	Caregiver continually supports and extends the child's actions throughout the recording.
Symbol Highlighting	Symbol highlighting refers to how a caregiver intentionally brings attention to symbols, such as spoken words, signs, labels, or pretend actions, during interaction with the child. The goal is to make these symbols more noticeable, meaningful, and easier for the child to learn. This can include repeating key words, emphasizing certain phrases, labeling objects, or pairing actions with clear verbal descriptions.	Caregiver rarely emphasizes symbols.	There is moderate number of attempts made to emphasize symbols made by the caregiver.	Caregiver continually attempts to focus the attention of the child on symbols.
Following in on Child's Focus	Following in on Child's Focus refers to how the caregiver joins and maintains the interaction by paying attention to what the child is already interested in and building the interaction around that focus. This means the caregiver observes what the child is looking at, playing with, or talking about, and then responds by joining that same topic or activity, rather than redirecting the child to something new.	Caregiver rarely follows in on child's focus.	Caregiver builds on the child's focus or a regular but not continual basis.	Caregiver continually joins and acts to sustain the child's interest.
Sustainability of Shared Topic	Sustainability of Shared Topic refers to how well the child and caregiver can keep focusing on the same topic, object, or activity over time during their interaction (e.g., the same toy, game, or conversation). It reflects whether the shared focus continues smoothly across multiple turns, rather than quickly shifting or breaking down.	Fleeting topics between caregiver and child.	Topics are maintained are maintained for moderate duration (e.g., sustaining a conversation or play for a few turns).	Topics are sustained for long periods of time during the recording.
Fluency and Connectedness	Fluency and Connectedness refers to how smooth, coordinated, and mutually responsive the interaction is between the child and caregiver. It captures whether their exchange flows naturally, with clear turn‐taking and logical links between each partner's actions or communication.	No interaction between caregiver and child is established.	Interaction lacks smoothness and appears largely dominated by either the caregiver or the child.	The interaction is fluid and balanced and often sustained between caregiver and child.
Expressive Language (child)	Expressive Language refers to the child's ability to produce and use language to communicate meaning to others during the interaction. This includes spoken words, word combinations, sentences, and other symbolic forms of language such as signs.	No expressive language.	The child produces many different intelligible words (≥ 10) during the recording with few or no word combinations.	The child is fluent and frequently uses sentences.

The specific JERI items used in this study were selected following detailed discussions with the developer. In addition, the first author spent 1 week in the developer's laboratory receiving training in JERI coding procedures, observing coding practices, and discussing study‐specific adaptations to ensure appropriate application of the measure to the current dataset.

For this study, 10 of the 17 available JERI items were selected based on their relevance to the study hypotheses. Item selection was guided by theoretical relevance to the proposed developmental model linking caregiver communicative behavior, joint engagement, and early socio‐cognitive development, rather than solely on empirical criteria. These included child‐centered engagement states (Total, Supported, Coordinated, and Symbol‐infused Joint Engagement), maternal behaviors (Scaffolding, Symbol Highlighting, and Following the Child's Focus), dyadic interaction measures (Sustainability of Shared Topic and Fluency and Connectedness) and child's Expressive Language. Although caregiver supportive behaviors are theoretically expected to support joint engagement, the JERI distinguishes between caregiver contributions to interaction and dyadic engagement states, permitting examination of their associations while recognizing their interdependence.

Importantly, joint engagement states are defined from the child's perspective, emphasizing that although the parent plays a critical role in shaping the interaction, the child's involvement and orientation toward the parent ultimately determine whether the parent becomes an integrated part of the child's play experience, either explicitly or implicitly.

Engagement states were first identified through systematic observation of the full interaction and then rated on a 7‐point scale that reflects both the proportion of time spent in each state and the qualitative features of engagement within that state, as defined by the JERI. Importantly, ratings capture not only duration but also the quality of engagement, coordination, and responsiveness between caregiver and child, as detailed in Table 2.

Total Joint Engagement captures the overall extent of the child's joint engagement with the caregiver around a shared object, event, or topic across the interaction. It provides a global measure of joint engagement, encompassing periods of both Supported and Coordinated Joint Engagement.

Supported Joint Engagement refers to periods of triadic engagement in which the child and caregiver are jointly engaged with a shared object or topic, with the caregiver providing support within the interaction and influencing the child's experience. This construct does not include situations in which the child and caregiver are merely attending to the same object or activity without the caregiver influencing the child's experience (e.g., parallel play). Ratings also reflect the quality of engagement, with a midpoint score indicating approximately one‐third of the interaction (around 3 min and 20 s) spent in supported joint engagement of moderate quality, or briefer but particularly high‐quality episodes.

Coordinated Joint Engagement describes interaction in which the child and caregiver share focus and the child actively coordinates attention between the object and the caregiver (e.g., through gaze shifts, gestures, or communicative acts). Ratings incorporate both time and quality, with a midpoint score indicating approximately one‐third of the interaction (around 3 min and 20 s) spent in Coordinated Joint Engagement of moderate quality or shorter but especially high‐quality episodes.

Symbolically Infused Joint Engagement captures episodes of supported or coordinated joint engagement that incorporate symbolic communication, such as spoken language, conventional gestures, signs, symbolic gestures (e.g., performing the finger movements for The Incy Wincy Spider while singing), or symbolic pretend play. These episodes extend beyond shared attention by involving symbols that represent objects, actions, or ideas beyond the immediate context. Ratings reflect both the frequency and the richness of symbolic communication demonstrated throughout the interaction.

Scaffolding refers to caregiver behaviors that support and extend the child's participation in interaction, play, or communication by providing appropriately calibrated assistance. This includes adjusting support to the child's level, prompting, modelling, and structuring activities to maintain engagement while encouraging increasing independence and complexity. Ratings reflect the extent and consistency of scaffolding across the interaction.

Symbol Highlighting refers to caregiver behaviors that intentionally draw attention to linguistic or symbolic information, such as emphasizing key words, labelling objects, repeating phrases, or pairing speech with actions to increase salience and meaning. Ratings reflect the frequency and continuity of these strategies.

Following in on the Child's Focus describes the caregiver's tendency to join and build on the child's existing focus of attention rather than redirecting it. This involves observing what the child is engaged with and responding in ways that sustain and extend that focus. Ratings reflect how consistently the caregiver aligns with and maintains the child's attentional focus over time.

Sustainability of Shared Topic refers to the extent to which caregiver and child maintain a consistent focus on the same topic, object, or activity across time. Higher ratings reflect longer and more coherent shared engagement, with a midpoint score indicating that topics are maintained for a moderate duration (several conversational or play turns across the interaction), and higher scores reflecting sustained topics across much or most of the session.

Fluency and Connectedness captures the smoothness, coordination, mutual responsiveness, and balance of the interaction. In the version of the JERI definition used in the present study, higher scores reflect balanced and reciprocal exchanges in which caregiver and child responses are clearly contingent on one another, contributing to a coherent and connected interactional flow. The detailed scoring anchors for this item are provided in Table 2.

Expressive Language refers to the child's demonstrated expressive language within the interaction. Ratings reflect both complexity and frequency of use, ranging from no expressive language to fluent sentence‐level speech. A midpoint score indicates the use of approximately 10 or more intelligible words with limited combinations, while higher scores reflect frequent and fluent use of multi‐word sentences. Full definitions and detailed scoring anchors for all items are provided in Table 2. The mean length of utterance was not calculated within this scoring system.

The first author received formal training in the JERI coding system at the developers’ laboratory, using gold‐standard videos and associated coding materials. Following this training, the first author coded a subgroup of WCHADS videos with guidance and support from staff at the developers’ laboratory. A second coder was subsequently trained by the first author using the same JERI coding materials, and coding guidance provided during the formal training. Although the second coder did not receive direct training from the JERI developers, they had access to the same coding framework used to establish the first author's competency.

To assess inter‐rater reliability, a subset of WCHADS videos (10%) was independently coded by the second coder. Given the ordinal nature of the JERI ratings, weighted Cohen's kappa coefficients with linear weighting were calculated for each domain. Reliability was good to excellent across categories, with weighted kappa values ranging from κw = .709 to .907. In addition, independent coders assigned ratings that were within one point of each other on the 7‐point scale for 91.7%–100% of observations across domains, indicating a high degree of coding agreement.

Coordinated Joint Engagement could not be reliably coded for six participants because portions of the video footage were of insufficient quality or visibility to permit accurate observation of coordinated attention. Other engagement states for these participants were sufficiently observable and were therefore reliably coded.

Given the conceptual and empirical overlap among several caregiver and dyadic interaction variables, composite constructs were specified to capture broader interactional dimensions. In particular, supportive behaviors (Scaffolding, Symbol Highlighting, and Following in on the Child's Focus) and dyadic interaction variables (Sustainability of Shared Topic and Fluency and Connectedness) were expected to reflect higher‐order constructs. These composites were theoretically motivated based on prior literature on caregiver support and interactional synchrony, with Principal Component Analysis used as a data‐reduction technique to derive parsimonious indices and reduce multicollinearity in subsequent analyses. Full details of Principal Component Analysis procedures are provided in the Statistical Analysis section.

## 6 CONFOUNDERS

### 6.1 Index of Multiple Deprivation

Socioeconomic status was determined using the revised Index of Multiple Deprivation (Noble et al., 2004). Postcode areas of England are ranked from the deprived (Index of Multiple Deprivation of 1) to the least deprived (Index of Multiple Deprivation of 32,482) based on seven domains of inequality (e.g., income deprivation, living environment deprivation, crime, etc.). All mothers were scored according to their area postal code.

### 6.2 Reading ability

Maternal reading ability was estimated using the Wechsler Test of Adult Reading (WTAR), a standardized measure of premorbid verbal intelligence. The WTAR requires pronunciation of 50 irregularly spelled words, with testing discontinued after 12 consecutive errors. Each correct response scores one point (maximum = 50), and raw scores are converted to age‐ and education‐adjusted standard scores (Holdnack, 2001). WTAR performance correlates strongly with verbal IQ, verbal comprehension, and full‐scale IQ, supporting the validity as a proxy measure of verbal cognitive ability (Strauss et al., 2006).

## 7 STATISTICAL ANALYSIS

All analyses were conducted in IBM SPSS Statistics (Version 27.0). Descriptive statistics summarized demographic, behavioral, and developmental characteristics, and bivariate correlations assessed associations and multicollinearity. To reduce multicollinearity and derive theoretically meaningful composite constructs, Principal Component Analysis were conducted on sets of highly correlated variables. Maternal supportive behaviors (Scaffolding, Symbol Highlighting, and Following in on Child's Focus) were entered into a Principal Component Analysis to derive a single latent factor representing maternal supportive behaviors. Similarly, Sustainability of Shared Topic and Fluency and Connectedness were entered into a separate Principal Component Analysis to derive a dyadic attunement factor. These composite variables were specified a priori based on theoretical considerations rather than derived purely empirically. Principal Component Analysis was used to confirm the extent to which these variables reflected shared underlying constructs and to provide parsimonious representations for use in mediation models, rather than to identify novel latent structures. In addition, individual variables were retained in initial regression analyses to examine specific associations, ensuring that findings were not solely dependent on composite measures. Linear regressions examined relationships between Communication Deviance and outcomes using unadjusted and adjusted models; adjusted models controlled for maternal age, reading ability, and Index of Multiple Deprivation. Mediation analyses tested indirect effects of Communication Deviance via maternal supportive behaviors. Mediation models were conducted using PROCESS (Hayes, 2013).

## 8 RESULTS

### 8.1 Characteristics of the sample and study variables

The mean age of the mothers was 27.58 years (standard deviation [SD] = 6.09), and they were predominantly White British (95.9%) reflecting the general demographics of the area in the UK (ONS, 2023). The mean reading ability score was 105.79 (*SD* = 6.53). The mothers were predominantly either married (40.4%) or in cohabitation with a partner (33.4%), with predominantly secondary school qualifications. Regarding the Index of Multiple Deprivation, the mean was 32.07 (*SD* = 20.1), reflecting high levels of deprivation, consistent with the study's catchment area. Table 3 also shows the means and standard deviations for both the Communication Deviance and the joint engagement states and related variables. Although mean ratings were relatively high across several joint engagement items, the full range of the scale (1–7) was utilized, and standard deviations indicated adequate variability, suggesting no substantial restriction of range or ceiling effects.

**TABLE 3:** Frequencies and descriptive statistics of the key variables in the study.

Variable	*N*	Mean (s.d.)/count (%)	Min.	Max.
Age	314	27.58 (6.09)	18	51
**Ethnicity**	White British	314	301 (95.9%)	
	Other		13 (4.1%)	
**Reading ability** (WTAR)	314	105.79 (6.53)	83	118
**Marital status**	Single		37 (11.8%)	
	Married		127 (40.4%)	
	Divorced		1 (0.3%)	
	Separated	313	2 (0.6%)	
	Cohabiting		105 (33.4%)	
	Partner lives elsewhere		40 (12.7%)	
	Other/Missing		2 (0.6%)	
**Education**	No qualifications		18 (5.7%)	
	CSE		4 (1.3%)	
	NVQ		98 (31.2%)	
	GNVQ		17 (5.4%)	
	A levels	308	114 (36.3%)	
	HNC/HND		2 (0.6%)	
	Undergraduate degree or above		15 (4.8%)	
	Other		40 (12.7%)	
	Missing		6 (1.9%)	
**Index of multiple deprivation**	314	32.07 (20.1)	3.99	79.73
	*32 weeks’ gestation*			
**Communication Deviance** (ratio)	287	0.96 (0.84)	0.00	5.30
**Communication Deviance** (Raw score)	287	4.60 (3.74)	0	28
**Word count**	287	583.69 (263.17)	25	1206
**Duration of speech sample** (min)	287	00:04:27 (00:01:08)	00:01:21	00:07:01
	*30 months postnatal*			
				
**Variable**	***N***	**Mean (s.d.)**	**Min**.	**Max**.
**Total Joint Engagement**	230	6.08 (1.11)	2	7
**Supported Joint Engagement**	230	5.84 (1.23)	1	7
**Coordinated Joint Engagement**	224	2.71 (1.29)	1	7
**Symbol‐infused Joint Engagement**	230	5.29 (1.56)	2	7
**Scaffolding**	230	5.13 (1.56)	1	7
**Symbol Highlighting**	230	5.37 (1.64)	1	7
**Following in on Child's Focus**	230	5.55 (1.50)	1	7
**Sustainability of Shared Topic**	230	5.33 (1.58)	1	7
**Fluency and Connectedness**	230	5.01 (1.69)	1	7
**Expressive Language (child)**	230	5.82 (1.30)	1	7

*Note*: All joint engagement ratings items are rated on a 7‐point scale (1–7). Higher scores indicate greater levels of the construct. Descriptive statistics include means, standard deviations, and observed ranges.

### 8.2 Joint engagement

To test whether Communication Deviance was a significant predictor of total time spent on joint engagement, an unadjusted linear regression was performed to test the bivariate relationship. In the unadjusted model, Communication Deviance was a significant predictor Total Joint Engagement, *B *= −.38, *SE* = .09, *β* = −.27, *t* = −4.18, *p *< .001. The model accounted for 7.1% of the variance, *R^2^* = .071, *F*(1228) = 17.44, *p* < .001. The same model, but adjusted for the mother's age, Index of Multiple Deprivation, and reading ability, was run. The overall model was significant, *F*(4225) = 5.64, *p* < .001, explaining 9.1% of the variance (*R^2 ^* = .091). Communication Deviance continued to significantly predict Total Joint Engagement, *B* = −.33, *SE* = .09, *β *= −.23, *t* = −3.49, *p* < .001 but maternal reading ability (*B* = .10, *p *= .416), age (*B* = .19, *p *= .151), and Index of Multiple Deprivation (*B* = −.001, *p *= .718) were non‐significant.

For Supported Joint Engagement, Communication Deviance was again a significant predictor, *B *= −.32, *SE* = .1, *β* = −.21, *t* = −3.16, *p *= .002. The unadjusted model accounted for 4.2% of the variance in Supported Joint Engagement, *R^2^ *= .042, *F*(1228) = 10.01, *p* = .002. The overall adjusted model was again significant, *F*(4225) = 3.28, *p* = .012, and explained 5.5% of the observed variance (*R^2 ^* = .055) with Communication Deviance predicting Supported Joint Engagement, *B* = −.29, *SE* = .11, *β *= −.18, *t* = −2.71, *p* = .007. Again, maternal reading ability (*B* = .02, *p *= .182) age (*B* = .01, *p *= .419), and Index of Multiple Deprivation (*B* = .003, *p *= .460) were non‐significant.

The same analysis for Coordinated Joint Engagement was repeated. Communication Deviance was again a significant predictor of this form of engagement, *B *= −.27, *SE* = .11, *β* = −.177, *t* = −2.69, *p <* .01. The unadjusted model accounted for 3.1% of the variance in Coordinated Joint Engagement, *R^2^ *= .031, *F*(1222) = 7.21, *p* = .008. The overall adjusted model was non‐significant, *F*(4219) = 2.03, *p* = .091, and explained 3.6% of the observed variance.

Finally, the analysis using Symbol‐infused Joint Engagement as the dependent variable yielded a positive unadjusted model, *F*(1228) = 19.55, *p* < .001, and explained 7.9% of the observed variance (*R^2 ^* = .079) with higher Communication Deviance scores associated with less Symbol‐infused Joint Engagement between the mother and the child, *B* = −.56, *β *= −.28, *t* = −4.42, *p* < .001. The adjusted model was significant, *F*(4225) = 6.28, *p* < .001, explaining 10% of the observed variance (*R^2^ *= .10). Communication Deviance remained a significant predictor, *B* = −.48, *SE* = .13, *β *= −.24, *t* = −3.65, *p* < .001, and maternal reading ability (*B* = .03, *p *= .071), age (*B* = .01, *p *= .535), and Index of Multiple Deprivation (*B* = −.001, *p *= .886) being non‐significant. Overall, higher maternal Communication Deviance was consistently associated with reduced time in all forms of joint engagement.

### 8.3 Maternal behaviors

For Scaffolding, the unadjusted model accounted for 17% of the variance, *R^2^ *= .17, *F*(1228) = 46.03, *p* < .001 and Communication Deviance was associated with less Scaffolding, *B *= −.81, *SE* = .12, *β* = −.41, *t* = −6.78, *p *< .001. The overall adjusted model was significant, *F*(4225) = 13.28, *p* < .001, and explained 19% of the observed variance (*R^2 ^* = .19) with Communication Deviance predicting reduced Scaffolding, *B* = −.73, *SE* = .12, *β *= −.37, *t* = −5.94, *p* < .001 and with the confounders being non‐significant, maternal reading ability (*B* = .02, *p *= .316), age (*B* = .03, *p *= .125) and Index of Multiple Deprivation (*B* = −.002, *p *= .642).

Communication Deviance was also a significant predictor of reduced Symbol Highlighting, *B *= −.77, *SE* = .13, *β* = −.37, *t* = −6.04, *p *< .001. The unadjusted model accounted for 14% of the variance, *R^2^ *= .14, *F*(1228) = 36.42, *p* < .001. The overall adjusted model was also significant, *F*(4225) = 9.88, *p* < .001, and explained 15% of the observed variance (*R^2 ^* = .15) with Communication Deviance again predicting reduced Symbol Highlighting, *B* = −.71, *SE* = .13, *β *= −.34, *t* = −5.301, *p* < .001 and with all the confounders being non‐significant, maternal reading ability (*B* = .02, *p *= .246), age (*B* = .01, *p *= .661), and Index of Multiple Deprivation (*B* = −.003, *p *= .609).

Finally, the unadjusted model with Following in on Child's Focus as the dependent variable, yielded significant findings with Communication Deviance being a negative predictor, *B *= −.76, *SE* = .12, *β* = −.398, *t* = −6.55, *p *< .001. The model accounted for 16% of the variance, *R^2^ *= .16, *F*(1228) = 42.93, *p* < .001. The adjusted model was significant, *F*(4225) = 12.55, *p* < .001, and explained 18% of the observed variance (*R^2 ^* = .18) with Communication Deviance predicting reduced Following in on Child's Focus, *B* = −.69, *SE* = .12, *β *= −.36, *t* = −5.78, *p* < .001 and with all the confounders being non‐significant, maternal reading ability (*B* = .01, *p *= .488), age (*B* = .03, *p *= .058) and Index of Multiple Deprivation (*B* = −.001, *p *= .804). Therefore, indicating that maternal Communication Deviance strongly predicts reductions in Scaffolding, Symbol Highlighting, and Following in on the Child's Focus. Given the high intercorrelations among these behaviors, subsequent analyses also examined a composite maternal supportive behaviors factor derived via Principal Component Analysis (see Methods).

### 8.4 Sustainability of shared topic and fluency and connectedness

The same analyses were repeated with Sustainability of Shared Topic and Fluency and Connectedness. Regarding Sustainability of Shared Topic, Communication Deviance was a significant predictor of reduced length of Sustainability of Shared Topic in the dyad, *B *= −.698, *SE *= .12, *β* = −.35, *t* = −5.62, *p *< .001. The unadjusted model accounted for 12% of the variance, *R^2^ *= .12, *F*(1228) = 31.596, *p* < .001. The overall adjusted model was significant, *F*(4225) = 9.46, *p* < .001, and explained 14% of the observed variance (*R^2 ^* = .14) with Communication Deviance as a significant predictor, *B* = −.61, *SE* = .13, *β *= −.305, *t* = −4.75, *p* < .001 and with all the confounders being non‐significant, maternal reading ability (*B* = .03, *p *= .095), age (*B* = .002, *p *= .895) and Index of Multiple Deprivation (*B* = −.005, *p *= .320).

In relation to Fluency and Connectedness, given that this item was highly correlated with Sustainability of Shared Topic (*r *= .83), Communication Deviance was unsurprisingly a significant predictor, *B *= −.71, *SE* = .13, *β* = −.33, *t* = −5.27, *p *< .001. The unadjusted model accounted for 11% of the variance (*R^2^ *= .109), *F*(1228) = 27.78, *p* < .001. The overall adjusted model was significant, *F*(4225) = 7.94, *p* < .001, and explained 12% of the observed variance (*R^2 ^* = .12) with Communication Deviance remaining a significant predictor, *B* = −.63, *SE* = .14, *β *= −.295, *t* = −4.53, *p* < .001 and with all the confounders being non‐significant, maternal reading ability (*B* = .02, *p *= .251), age (*B* = .02, *p *= .422), and Index of Multiple Deprivation (*B* = −.003, *p *= .663). Given the high correlation between Sustainability of Shared Topic and Fluency and Connectedness (*r *= .83), subsequent analyses also examined a composite Dyadic Attunement factor derived via Principal Component Analysis (see Statistical Analysis).

### 8.5 Expressive Language

Finally, to examine whether maternal Communication Deviance at 32 weeks’ gestation predicted the child's Expressive Language at 30 months a subsequence analysis was run. The unadjusted model was non‐significant, *F*(1228) = 1.57, *p* = .211, and Communication Deviance did not predict Expressive Language in the child, *B* = −.14, *SE* = .11, *β *= −.08, *t* = −1.25, *p* = .211. Table 4 shows all linear regressions using Communication Deviance as the predicting variable.

**TABLE 4:** Linear regressions with Communication Deviance as the predicting variable.

Dependent variables (30 months)		Unadjusted model		Adjusted model^1^	
	*N*	Standardized coefficient	*p*‐value	Standardized coefficient	*p*‐value
Total Joint Engagement	229	−0.267	< 0.001	−0.231	< 0.001
Supported Joint Engagement	229	−0.205	0.002	−0.183	0.007
Coordinated Joint Engagement	223	−0.177	0.008	−0.167	0.016
Symbol‐infused Joint Engagement	229	−0.281	< 0.001	−0.241	< 0.001
Scaffolding	229	−0.410	< 0.001	−0.371	< 0.001
Symbol Highlighting	229	−0.371	< 0.001	−0.340	< 0.001
Following in on Child's Focus	229	−0.398	< 0.001	−0.363	< 0.001
Sustainability of Shared Topic	229	−0.349	< 0.001	−0.305	< 0.001
Fluency and Connectedness	229	−0.330	< 0.001	−0.295	< 0.001
Expressive Language (Child)	229	−0.083	ns	−0.050	ns

^1^Regression model adjusted for mother's age, reading ability, and Index of Multiple Deprivation.

### 8.6 Testing mediation

As specified in the Methods, Principal Component Analysis was used to derive composite variables for maternal supportive behaviors and dyadic attunement. The supportive behaviors variables were strongly intercorrelated (all *r* > .54), with adequate sampling adequacy (KMO = .698) and a significant Bartlett's test (*p* < .001). A single factor was extracted, loading most strongly on supportive behaviors, and was used in subsequent mediation analyses. A similar procedure was applied to dyadic attunement variables (*r* = .83; KMO = .50; Bartlett's *p* < .001), yielding a single factor.

Separate mediation analyses were conducted using the PROCESS macro in SPSS. Communication Deviance was entered as the predictor, maternal supportive behaviors as the mediator, and joint engagement variables (Total Joint Engagement, Supported Joint Engagement, Coordinated Joint Engagement, and Symbol‐infused Joint Engagement), and the dyadic attunement factor as dependent variables. Maternal age, reading ability, and Index of Multiple Deprivation were included as covariates. Analyses were conducted separately for clearer interpretation as some of the scores on joint engagement inherently trade off against each other. The significance of indirect effects was assessed using 95% bias‐corrected bootstrapped confidence intervals (CI) based on 5000 bootstrap samples. In the analyses, Communication Deviance was again a significant predictor of maternal supportive behaviors factor (*B* = −.48, *SE* = .07, 95% *CI* [−.62, −.34]). The maternal supportive behaviors factor was then tested as a mediator of the relationship between Communication Deviance and the different outcomes. Firstly, the maternal supportive behaviors factor significantly predicted greater Total Joint Engagement (*B* = .80, *SE* = .07, 95% *CI* [.66, .93]). The indirect effect of Communication Deviance on Total Joint Engagement via the supportive behaviors factor was significant (*B *= −.38, *BootSE* = .07, 95% *CI* [−.55, −.26]), with the direct effect being non‐significant (*B* = .06, *SE* = .08, 95% *CI* [−.10, .21]), indicating full mediation; therefore, suggesting that the supportive behaviors factor fully mediates the association between maternal Communication Deviance and Total Joint Engagement. Secondly, the maternal supportive behaviors was a significant predictor of Supported Joint Engagement (*B* = .74, *SE* = .08, 95% *CI* [.58, .91]). The indirect effect of Communication Deviance on Supported Joint Engagement via the supportive behaviors factor was significant (*B* = −.36, *BootSE* = .08, 95% *CI* [−.52, −.23]), with the direct effect being non‐significant (*B *= .07, *SE* = .10, 95% *CI* [−.13, .27]), indicating full mediation; therefore, reductions in supportive behaviors accounted for the association between Communication Deviance and Supported Joint Engagement. Furthermore, the maternal supportive behaviors factor significantly predicted higher Coordinated Joint Engagement (*B* = .51, *SE* = .10, 95% *CI* [.32, .70]). The indirect effect of Communication Deviance on Coordinated Joint Engagement via supportive behaviors was significant (*B* = −.24, *BootSE* = .06, 95% *CI* [−.38, −.14]), with the direct effect being non‐significant (*B* = −.03, *SE* = .12, 95% *CI* [−.26, .20]), indicating full mediation; therefore, suggesting that supportive behaviors may be a mechanism through which Communication Deviance may influence Coordinated Joint Engagement. Additionally, the maternal supportive behaviors factor significantly predicted higher Symbol‐infused Joint Engagement (*B* = .95, *SE *= .10, 95% *CI* [.76, 1.14]). The indirect effect of Communication Deviance on Symbol‐infused Joint Engagement via the supportive behaviors factor was significant (*B *= −.46, *BootSE* = .09, 95% *CI* [−.66, −.31]), with the direct effect being non‐significant (*B* = −.02, S*E* = .12, 95% *CI* [−.26, .22]), indicating full mediation; therefore, suggesting that maternal supportive behaviors mediates the impact of Communication Deviance on Symbol‐infused Joint Engagement. Maternal supportive behaviors significantly predicted the higher dyadic attunement factor too (*B* = .75, *SE* = .06, 95% *CI* [.64, .86]). The indirect effect of Communication Deviance on dyadic attunement via supportive behaviors was significant (*B* = −.36, *BootSE* = .06, 95% *CI* [−.49, −.26]), with the direct effect being non‐significant (*B* = −.04, *SE* = .07, 95% *CI* [−.17, .09]), indicating full mediation. This suggests that maternal supportive behaviors may underpin the association between higher Communication Deviance in the mother and poorer conversational fluency and topic sustainability in the dyad. Lastly, maternal supportive behaviors significantly predicted higher Expressive Language in the child (*B* = .35, *SE* = .1, 95% *CI* [.15, .55]). The indirect effect of Communication Deviance on Expressive Language via supportive behaviors was significant (*B* = −.17, *BootSE* = .05, 95% *CI* [−.28, −.07]), with the direct (*B *= .09, *SE* = .12, 95% *CI* [−.15, .32]), and total effect (*B* = −.08, *SE* = .11, 95% *CI* [−.31, .14]), being both non‐significant indicating indirect‐only mediation. Though Communication Deviance did not directly predict Expressive Language, the indirect effect through supportive behaviors highlights the developmental importance of maternal support. Overall, maternal supportive behaviors was consistently a significant mediator for all forms of joint engagement, dyadic attunement, and Expressive Language. Standardized coefficients for all relationships are summarized in Figure 1.

**FIGURE 1:**
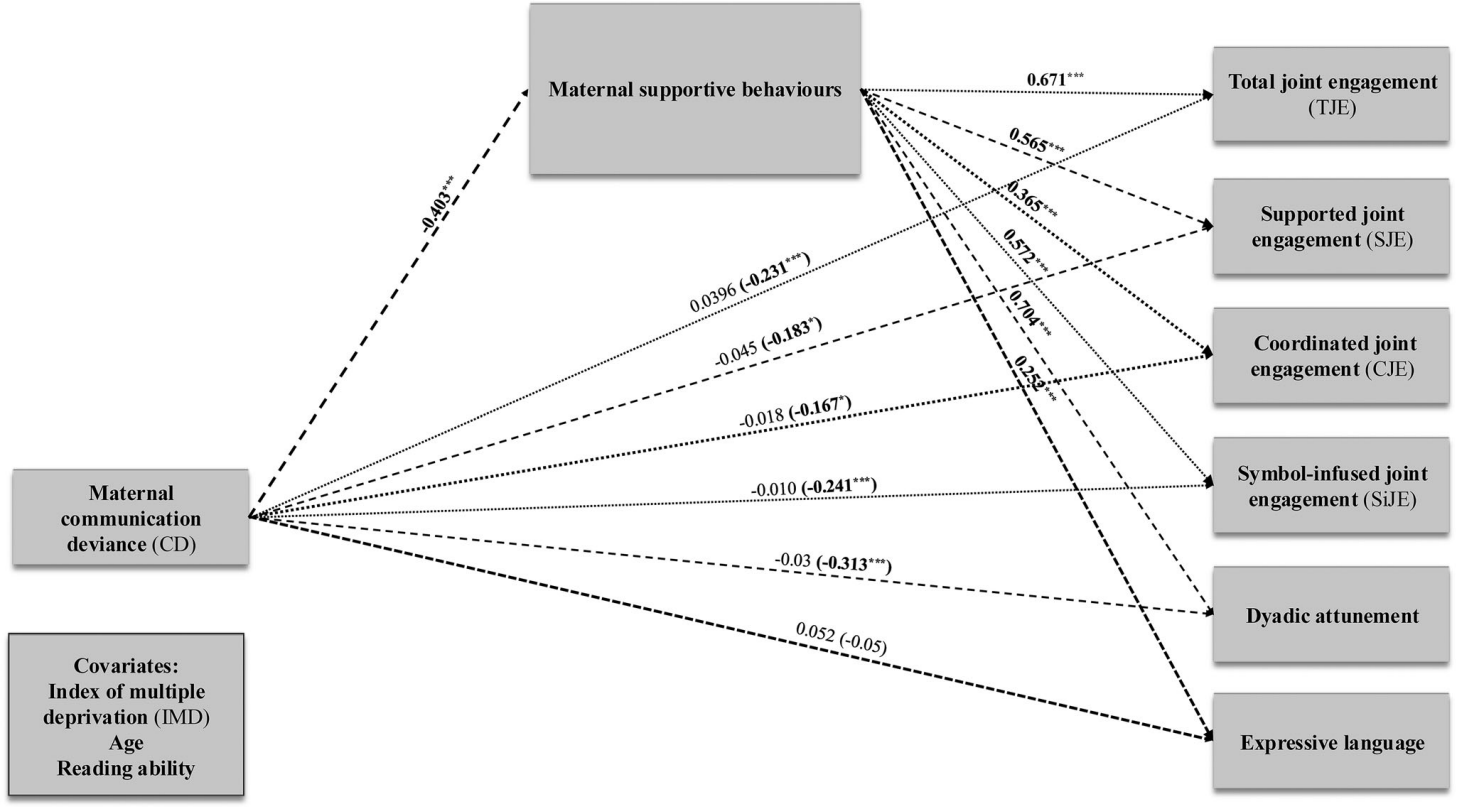
Path coefficients, direct effects, and indirect effects (standardized coefficients).

## 9 DISCUSSION

This study extends research on parental Communication Deviance by providing new empirical evidence regarding its developmental significance. Specifically, our findings contribute to understanding the role of parental Communication Deviance in developmental pathways and its potential implications for child outcomes. It was hypothesized that early caregiver communicative disturbances disrupt joint engagement in infancy, potentially cascading into broader socio‐cognitive difficulties. In a longitudinal cohort, higher maternal Communication Deviance at 32 weeks of pregnancy robustly predicted lower joint engagement at 30 months postpartum across domains (Total, Supported, Coordinated, and Symbol‐infused Joint Engagement states). These associations held after controlling for maternal age, reading ability, and Index of Multiple Deprivation. The findings underscore the enduring influence of maternal communicative style on mother‐child interaction quality. Given that Communication Deviance levels in this cohort were lower than typically reported in high‐risk samples, the restricted range has likely attenuated the effect sizes, so the observed associations may underestimate the strength of the underlying relationships.

### 9.1 Communication Deviance as a predictor of joint engagement

Results suggest that Communication Deviance may compromise the foundational processes of dyadic interaction. Communication Deviance predicted not only less total time spent by the dyad on joint engagement but also reduced Supported and Coordinated Joint Engagement.

The association between higher maternal Communication Deviance and lower Symbol‐infused Joint Engagement suggests that communicative disturbances may reduce opportunities for symbolically enriched shared engagement between caregiver and child. Because Symbol‐infused Joint Engagement is a dyadic construct that incorporates both caregiver and child contributions, this finding may reflect differences in the extent to which symbolic communication is incorporated into shared interaction, rather than deficits in children's symbolic abilities per se. Effects on Symbol‐infused Joint Engagement suggest that higher maternal Communication Deviance may be associated with fewer opportunities for symbolically enriched shared engagement between caregiver and child. Because Symbol‐infused Joint Engagement reflects a dyadic process involving the incorporation of language, symbols, and pretend play into shared interaction, these findings may indicate that communicative disturbances reduce the extent to which symbolic communication becomes integrated into joint engagement episodes. These findings align with theoretical accounts suggesting that shared meaning‐making and symbolic reference rely heavily on the caregiver's capacity to structure and sustain affectively attuned, communicatively rich interactions (Adamson et al., 2004; Hobson, 2005).

### 9.2 Mediating role of maternal supportive behaviors and attunement

Mediation analyses revealed that the effect of Communication Deviance on joint engagement was significantly mediated by maternal supportive behaviors. Communication Deviance was strongly associated with reductions in these behaviors, which in turn predicted less time spent by the dyad on joint engagement and poorer dyadic attunement. These findings are consistent with research finding that maternal supportive behaviors is a key process where the caregiver supports the development of attention, language, and emotion regulation (Brandes‐Aitken et al., 2020; Schatz et al., 2022). The direct effect of maternal Communication Deviance on joint engagement became non‐significant when supportive behaviors was included in the regression model, underscoring supportive behaviors as a possible key behavioral mechanism linking maternal Communication Deviance and early engagement. It is important to note that these constructs are conceptually distinct. Supportive behaviors reflect caregiver‐driven behaviors that structure and support the child's participation in interaction, whereas joint engagement captures the emergent dyadic state of shared attention and reciprocity. The strong association between these constructs is therefore theoretically expected, as supportive behaviors are thought to support and sustain joint engagement episodes rather than constitute them. Nevertheless, because both constructs were coded from the same interaction and derived from the same observational system, the mediation findings should be interpreted cautiously as evidence that supportive behaviors statistically account for the association between Communication Deviance and joint engagement, rather than as definitive evidence of a temporally distinct causal process. Moreover, given the substantial intercorrelations among items, the variance explained by individual models should be interpreted with caution, as these behaviors reflect overlapping aspects of broader interactional constructs.

Moreover, Communication Deviance was a robust predictor of dyadic attunement, operationalized as Sustainability of Shared Topic and Fluency and Connectedness. Findings suggest that maternal Communication Deviance may disrupt communicative clarity and the temporal and emotional synchrony underpinning high‐quality mother–child interactions (Hobson & Hobson, 2011). This synchrony of attunement is thought to form the basis of shared intentionality and reciprocal engagement, central tenets of sociocultural and enactivist models of development (Racine & Carpendale, 2007; Trevarthen & Aitken, 2001).

### 9.3 Communication Deviance as an early environmental risk factor

Findings converge and extend prior research linking maternal Communication Deviance to child psychopathology, highlighting potential developmental pathways through which Communication Deviance may operate during the early vulnerability period. Extending prior longitudinal research linking maternal Communication Deviance to adolescent psychopathology (Tienari et al., 1985; Wahlberg et al., 1997), the present findings show associations with disrupted mother‐child interaction from as early as 30 months postpartum; therefore, supporting that Communication Deviance is not a downstream correlate of offspring vulnerability, or high‐risk status, but may function as an independent environmental risk factor shaping early developmental pathways. Importantly, Communication Deviance did not predict expressive language in the child at 30 months. It is possible that any direct effects of Communication Deviance on language development may emerge later, when more advanced and fluent language abilities are expected. The mean Expressive Language rating in the present sample indicated that most children were producing multiple intelligible words and simple word combinations but had not yet reached the highest levels of fluent sentence production captured by the scale. Furthermore, the JERI Expressive Language item primarily reflects the quantity and complexity of language production rather than the coherence, organization, clarity, or pragmatic appropriateness of language. Given that Communication Deviance is theoretically linked to difficulties in shared meaning and communicative coherence, its influence may be more readily detectable in later emerging aspects of language and communication than in early expressive language production alone.

### 9.4 Implication and future directions

The findings suggest that Communication Deviance in the caregiver may be associated with disruption of early joint engagement and shared symbolic experiences with the child, which are critical for healthy socio‐cognitive development. These results point to potential targets for preventative work with families at risk or parents with diagnoses of severe and enduring mental illness. This is an area of work which is neglected (Davidsen et al., 2015; Harder et al., 2015). Training programs focused on enhancing maternal responsiveness and supportive behaviors during joint engagement may offer a viable pathway for mitigating the developmental impact of Communication Deviance, especially in families with known risk factors. Interventions could prioritize enhancing maternal responsiveness and supportive behaviors during early joint engagement episodes. Practical approaches could include structured coaching on following in on the child's focus, symbol highlighting, and supportive behaviors strategies, particularly for mothers with known psychosocial or mental health risk factors or diagnoses. Future research could use micro‐behavioral and sequential analytic approaches to examine how Communication Deviance influences the moment‐to‐moment dynamics of parent‐child interaction. For example, are interactions less mutually contingent, or do parents respond less contingently to children's communicative bids and symbolic play? In addition, future studies should also include postpartum assessments of Communication Deviance to examine whether communicative style remains stable once the child becomes an active partner, and to clarify the developmental timing of its effects. It would also be interesting in future research to antenatally examine maternal mental health and trauma history as potential covariates and predictors of Communication Deviance, given their likely influence on communicative style and early interactional processes. Another avenue will be to explore the extent to which relational disruptions mediate long‐term outcomes in social cognition, school readiness, and psychopathology. Given that Communication Deviance is not specific to schizophrenia‐spectrum conditions and appears across various mental health risks (Hirsch & Leff, 1971; Miklowitz et al., 1991), these findings support its conceptualization as a transdiagnostic dimensional marker of communicative disturbance. Further investigation into neurobiological correlates and genetic markers (Levy et al., 2010) of Communication Deviance, may clarify how genetic and environmental factors co‐produce developmental risk. Additionally, based on Lavoie and colleagues’ research into social cognition deficits in first‐degree relatives of individuals with schizophrenia (Lavoie et al., 2013, 2014), it may be valuable to examine associations between social cognition, mentalization, and Communication Deviance, and socio‐cognitive development as another potential early environmental risk factor in the emergence of mental illness. Future research should also examine these processes in culturally and linguistically diverse samples to determine whether these associations vary across different caregiving contexts. Finally, future research using micro‐temporal or sequential analytic approaches would be valuable in determining whether supportive behaviors temporally precede and predict transitions into joint engagement states, thereby clarifying the directionality of these effects.

### 9.5 Limitations

Some limitations warrant consideration. Our sample was drawn from a relatively homogeneous sample in a specific geographic area, potentially limiting generalizability. As the sample was almost entirely White British, cultural and linguistic homogeneity may have shaped expression of Communication Deviance, supportive behaviors, and joint engagement, therefore further limiting generalizability to more diverse populations. Moreover, while Communication Deviance was measured prospectively and before the child's birth, all other constructs were assessed concurrently at 30 months, which limits causal inference.

Another limitation relates to the use of Five‐Minute Speech Samples as a methodology to elicit speech samples. Although this has been used previously to assess Communication Deviance, it is possible that the 5‐min sample methodology may underestimate the true variability and contextual expression of Communication Deviance in daily life. It would be interesting in future studies to use more conversational speech samples. Regarding the joint engagement rating approach, although the JERI provides detailed scoring guidance and behavioral examples (Adamson et al., 2020), observational ratings inevitably involve a degree of coder judgement when integrating interactional behaviors across an entire session into global ratings. Consequently, subtle differences in interpretation between coding teams cannot be entirely excluded despite formal training and good inter‐rater reliability.

Additionally, Communication Deviance was assessed antenatally to minimize reverse causality, as maternal communication could be influenced by the child postnatally. Whilst Communication Deviance shows good temporal stability (Nugter et al., 1997; Wahlberg et al., 2001), the expression may shift once the child becomes an active communicative partner. The absence of a post‐partum assessment further limits conclusions about developmental timing and warrants consideration as a limitation. Another limitation is that formal assessment of first‐language status was not available, although Communication Deviance coding focuses on communicative coherence rather than linguistic proficiency.

A consideration when interpreting the JERI findings concerns the coder training procedure. Although the first author received formal training at the JERI developers’ laboratory using gold‐standard videos and subsequently coded the WCHADS material with support from laboratory staff, the second coder was trained by the first author rather than directly by the JERI developers. The second coder nevertheless had access to coding materials used during the first author's training, and inter‐rater reliability was assessed through independent double‐coding. This procedure provides support for the consistency of the coding; however, the shared training pathway means that some influence arising from common training or coder expectations cannot be entirely excluded. This is relevant when interpreting the relatively high JERI scores observed in the present sample.

An additional consideration concerns the relatively high mean ratings observed across several JERI domains, particularly caregiver supportive behavior items and Sustainability of Shared Topic. Although the full range of ratings was used and inter‐rater reliability was good to excellent, the pattern of scores may suggest that some interactional behaviors were coded more liberally than intended in the original JERI framework. This possibility is difficult to evaluate directly because no cross‐site calibration with contemporary JERI coding teams was available. If present, such differences would likely influence the absolute ratings assigned to interactions rather than the pattern of associations observed within the current sample. Accordingly, findings should be interpreted primarily in terms of relative differences between dyads rather than as estimates of absolute levels of engagement or caregiver support.

A further consideration concerns the fact that maternal supportive behaviors and Joint Engagement were derived from observations of the same interaction. Although these constructs are related, they are conceptually distinct within the JERI framework. Supportive behaviors reflect caregiver actions that may facilitate engagement, whereas Joint Engagement is coded from the child's perspective and requires the child to acknowledge the caregiver as a social partner in the shared activity. Thus, caregiver efforts alone are insufficient to determine Joint Engagement ratings. Nevertheless, because both constructs were rated from the same interactional context, some degree of shared observational variance cannot be entirely excluded. The mediation findings should therefore be interpreted as identifying a plausible explanatory pathway rather than providing definitive evidence of a temporally independent causal mechanism.

## 10 CONCLUSION

These findings enrich understanding of how subtle early environmental risk factors may shape socio‐cognitive development in the context of heightened familial vulnerability. Maternal Communication Deviance was directly associated with reduced joint engagement across multiple dimensions and with lower scaffolding, symbol highlighting, and dyadic attunement at 30 months. Mediation analyses further showed that these effects were explained by maternal supportive behaviors, demonstrating the role of these behaviors as a key mechanism linking Communication Deviance to early interactional quality.

## Pre‐registration statement

This study was not pre‐registered. The research involved secondary data analysis of observational data collected as part of the Wirral Child Health and Development Study (WCHADS), a longitudinal cohort study. At the time of data collection, pre‐registration was not standard practice for secondary analyses of this kind. Further information about the WCHADS cohort can be found at: https://www.cataloguementalhealth.ac.uk/?content=study&studyid=WCHADS and https://www.liverpool.ac.uk/population‐health/research/groups/first‐steps/

## FUNDING STATEMENT

This study was supported by the UK Medical Research Council (Grant G0400577 to Jonathan Hill and Helen Sharp) and the Fundação para a Ciência e a Tecnologia (Grant SFRH/BD/77,379/2011 to Paulo de Sousa). Additional support was provided by Wirral University Teaching Hospital NHS Foundation Trust, Cheshire and Wirral Partnership NHS Foundation Trust, Wirral Community NHS Foundation Trust, the NIHR Biomedical Research Centre for Mental Health at South London and Maudsley NHS Foundation Trust, and King's College London.

## CONFLICT OF INTEREST STATEMENT

All authors declare that there are no competing interests.

## Data Availability

The data that support the findings of this study are available from the corresponding author upon reasonable request.
